# The fast contribution of visual-proprioceptive discrepancy to reach aftereffects and proprioceptive recalibration

**DOI:** 10.1371/journal.pone.0200621

**Published:** 2018-07-17

**Authors:** Jennifer E. Ruttle, Bernard Marius ‘t Hart, Denise Y. P. Henriques

**Affiliations:** 1 Centre for Vision Research, York University, Toronto, Canada; 2 Department of Psychology, York University, Toronto, Canada; 3 School of Kinesiology and Health Science, York University, Toronto, Canada; University of Alberta, CANADA

## Abstract

Adapting reaches to altered visual feedback not only leads to motor changes, but also to shifts in perceived hand location; “proprioceptive recalibration”. These changes are robust to many task variations and can occur quite rapidly. For instance, our previous study found both motor and sensory shifts arise in as few as 6 rotated-cursor training trials. The aim of this study is to investigate one of the training signals that contribute to these rapid sensory and motor changes. We do this by removing the visuomotor error signals associated with classic visuomotor rotation training; and provide only experience with a visual-proprioceptive discrepancy for training. While a force channel constrains reach direction 30^o^ away from the target, the cursor representing the hand unerringly moves straight to the target. The resulting visual-proprioceptive discrepancy drives significant and rapid changes in no-cursor reaches and felt hand position, again within only 6 training trials. The extent of the sensory change is unexpectedly larger following the visual-proprioceptive discrepancy training. Not surprisingly the size of the reach aftereffects is substantially smaller than following classic visuomotor rotation training. However, the time course by which both changes emerge is similar in the two training types. These results suggest that even the mere exposure to a discrepancy between felt and seen hand location is a sufficient training signal to drive robust motor and sensory plasticity.

## Introduction

In classic visuomotor training participants are able to explore the nature of the visual perturbation and alter their movements to compensate and achieve the target. Little training with altered visual feedback of the hand is required to lead to robust compensatory changes in reach direction, i.e., adaptation, within only 20–30 trials [1]. Work from our lab suggests that at least two types of training signals drive adaptation, one being the usual error-based signals [2,3] and the other being a visual-proprioceptive discrepancy signal regarding state estimates of the hand [4,5]. Both of these training signals are present during classic visuomotor training with volitional movements. Prior to volitional movements being executed, a motor plan is created and with it, expected sensory consequences [4,6,7]. When the cursor moves in an unexpected direction during rotated training, the difference between expected and actual movement consequences produce the visuomotor “sensorimotor error-based” signal mentioned above, which is associated with model-based learning [8]. The visual-proprioceptive discrepancy stems from the mismatch between the cursor or visual hand representation and the actual, felt, hand position [4]. This discrepancy provides a training or error signal that has been shown to drive recalibration of felt hand position and motor output [5,9,10]. Both these training signals are thought to drive primarily implicit (model-based) changes that occur during and following training. A combination of processes, based on these error and discrepancy signals contribute to generate the changes in movement which constitute adaptation.

Although some work has demonstrated the time course of explicit learning during training [11,12], we know little about how quickly supposedly implicit changes emerge. One measure of implicit learning is the persistent change in reach direction produced after the perturbation is removed, known as reach aftereffects [13], which we will focus on here. Following classic visuomotor rotation training, aftereffects are shown to be approximately 40–80% of the imposed distortion, regardless of rotation size and direction[14–20]. Aftereffects are not only robust immediately following classic training but also remain 24 hours later [15]. It has recently been shown, that these reach aftereffects can also emerge quite quickly; within 6 classic training trials, reach deviations were found to be 30% of the applied rotation [21,22]. Thus, reach aftereffects are a consistent and meaningful measure in capturing implicit motor adaptation, not only after, but throughout training.

Training with a kinematic or dynamic perturbation has been shown to cause not only aftereffects, but also changes in the perceived direction or position of the trained hand, what we call proprioceptive recalibration [16–18,23]. Following training with these perturbations, the shift in perceived location or motion of the unseen hand is in the direction of the distortion experienced and usually 15–25% of the size of the distortion [15,20,24]. These proprioceptive shifts are robust, consistently emerging regardless of the nature and size of visuomotor distortion [14,22–24] and have also been found to remain for at least 24 hours after training [15,17]. Our lab and collaborators have proposed that such proprioceptive changes may partly contribute to or even account for changes in reach aftereffects [4,18]. It is clear that proprioception is an important sensory signal to motor learning, and that proprioceptive recalibration is just as consistent as reach aftereffects.

Recent studies have started to explore the rate or time course by which proprioceptive changes emerge during training. Some studies have shown changes in felt hand motion that occur after 70 reach training trials, using a two-alternative forced choice method (2-AFC) [16,21]. Recently, we have shown that when using a quicker measure of proprioceptive recalibration, one that does not require 40+ 2-AFC trials, these trial-by-trial changes occur surprisingly quickly, after only 6 classic reach-training trials [22]. This rapid recalibration achieves full asymptotic levels in only a dozen more trials. We found these fast sensory changes to be accompanied by significant reach aftereffects after only 6 training trials, and unlike recalibrated proprioception the reach aftereffects grow much larger throughout training [21,22]. While these results highlight the speed at which these changes take place, it is unclear how much of this is driven merely by the visual-proprioceptive discrepancy, compared to any traditional sensorimotor-related error or training signals.

While both visuomotor errors and the visual-proprioceptive discrepancy may contribute to motor adaptation, their respective contributions are hard to separate. Classic visuomotor training allows participants full control of movements and both training signals are available. By restricting participant’s volitional control over their movements [9,25,26], we can eliminate the sensorimotor error signal to isolate the effects of visual-proprioceptive discrepancy on learning. We call this “exposure training” and even without volitional movements it has been shown to facilitate subsequent adaptation with the same or opposite hand [26,27]. Our lab has shown it consistently leads to reach aftereffects as well as changes in felt hand position [9]. Reach aftereffects following exposure training are robust yet somewhat smaller (only ~10–20% of distortion) than those produced following classic visuomotor training [5,9,10,25]. However, changes in felt hand position following exposure training are comparable to those observed following classic-volitional training [5,9,10,25]. In summary, exposure training produces reach aftereffects without volitional movements, even though it relies on visual-proprioceptive discrepancies, a training signal not usually considered in motor learning.

Exposure training can be fully passive, e.g., when a robot produces the movements, or it can be semi-active, when the participant produces the movement, but the movement direction is dictated by the apparatus. In either case there are no visuomotor errors, since the cursor goes straight to the target, but it is possible that semi-active exposure training could still evoke an error-based training signal contributing to adaptation. However we observed that semi-active and fully passive exposure training produce equivalent reach adaptation [9]. This indicates that it is the error in movement *direction* of the cursor that is most important to the visuomotor error signal. Regardless of which version of semi-active or fully passive exposure training used, taking away the sensorimotor error signals allows investigating the contribution of the visual-proprioceptive discrepancy to motor learning. To summarize, exposure training produces adaptation without volitional movements either fully passive or semi-active, even though it does not rely on visuomotor errors, but on visual-proprioceptive discrepancies.

Despite several studies demonstrating that visual-proprioceptive discrepancy as a training signal leads to robust changes in reaches and hand proprioception, no one has measured how quickly this signal can drive such changes. Thus the first two goals of this study are to characterize the rate and extent by which reaches and hand proprioception change during exposure training. This was accomplished by measuring reach aftereffects and proprioceptive localizations after every six exposure-training trials. The results should provide insight into the dynamic role that visual-proprioceptive discrepancy plays in motor learning. While we expect to see significant reach aftereffects and proprioceptive recalibration, as in previous studies [5,9], the aim here is to capture how quickly these changes emerge during training. Next, we wanted to compare the time course of these changes with those produced during classic visuomotor training, where both error-based signals and visual-proprioceptive discrepancy signals are available. Thus, we designed the current exposure study to match the procedure from our previous study using classic visuomotor training [22] to allow us to compare the rate by which reach aftereffects and proprioceptive recalibration develop during training. This should allow us to gauge the contribution of visual-proprioceptive discrepancies to the sensory and motor changes associated with motor learning.

## Methods

### Participants

The experiment included 19 (mean age = 23.6, range = 18–47, males = 13) right-handed, healthy adults. Participants were naïve to the purpose of the study and were given course credit for participation. All participants had normal or corrected to normal vision and were free from any physical or neurological conditions. All participants provided prior written informed consent and the study was approved by the York Human Participants Review Subcommittee.

### Apparatus

**Fig 1:**
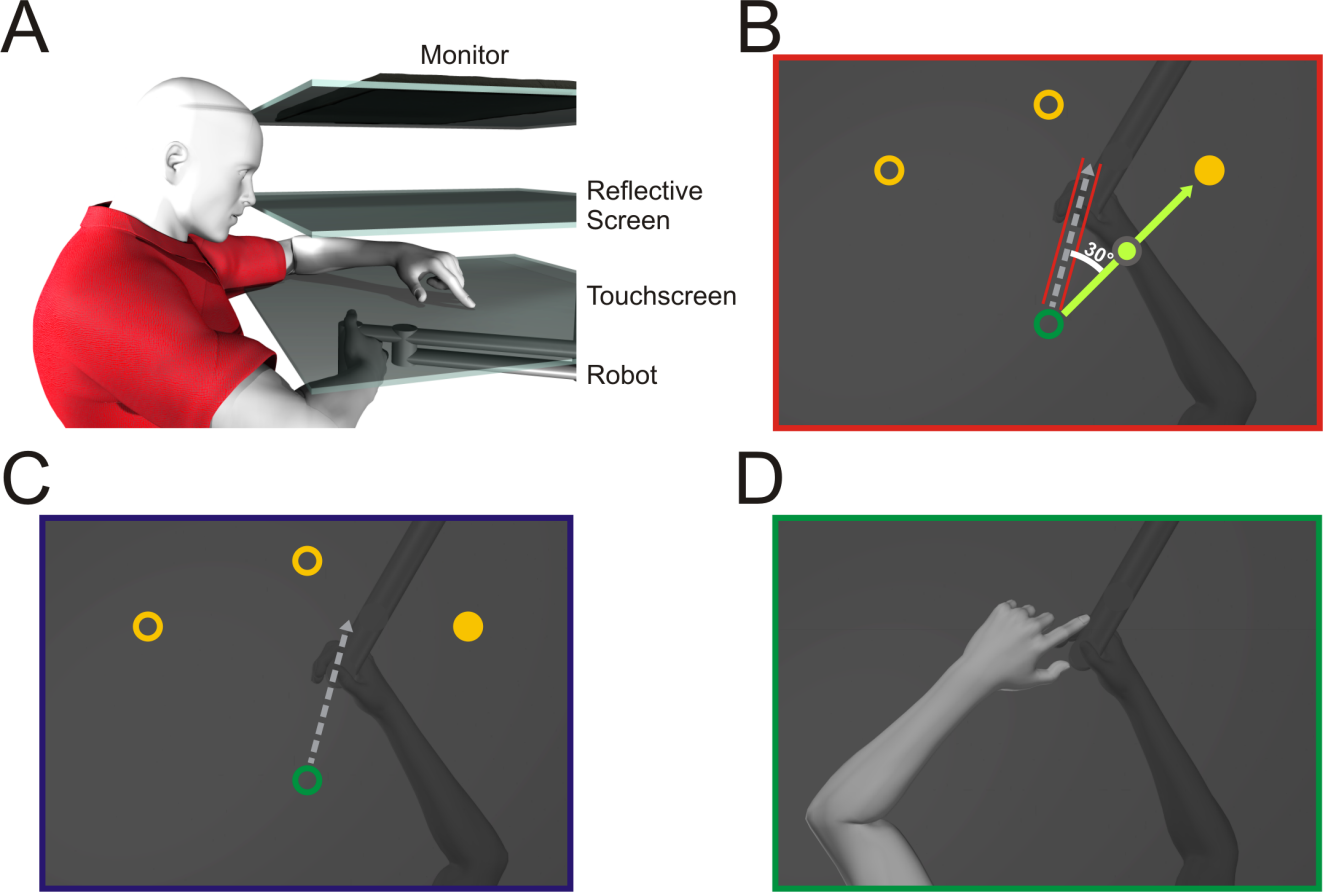
Experimental setup and design. **A:** Side view of the experimental set-up. The top layer is the monitor, middle layer is the reflective screen, and the bottom layer is the touchscreen. The robot is depicted beneath with the participants’ right hand grasping it. **B-D:** Top views of task specific set-ups. **B**: **Training task.** The home position is represented by a green circle with a 1 cm diameter; located approximately 20 cm in front of the subject and not visible during the trial. Targets are represented by yellow circles with a 1 cm diameter located 12 cm radially from the home position at 60°, 90° and 120°. The target was visible for 250 ms, after which it disappeared and participants moved their right hand along the constrained force channel (shown in red) to its remembered location. During rotated exposure training the constrained hand path was rotated 30° CCW from target with respect to the start location: **C No-cursor reach task.** The same target locations were used as during training. The participant would freely reach, without the force channel that was present during exposure training trials and without the cursor or any other visual feedback of the hand. **D: Localization task.** In the proprioceptive localization task, the robot passively moved the unseen, right trained hand to one of the three target locations. The participants then used the index finger of their left untrained, visible, hand to indicate the felt location of the right hand, specifically the thumb.

A view of the experimental set-up is provided in Fig 1. Participants sat in a chair that could be adjusted in height and distance from the display so that they could comfortably see and reach to each of the target locations presented on a reflective screen (Fig 1A). With their right hand, participants held onto a vertical handle on a two-joint robot manipulandum (Interactive Motion Technologies Inc., Cambridge, Ma, USA) such that their thumb rested on top of the modified handle. The reflective screen was mounted on a horizontal plane 14.5 cm above the two-joint robotic arm. Visual stimuli were projected from a monitor (Samsung 510 N, refresh rate 60 Hz) located 36 cm above the robotic arm such that images displayed on the monitor appeared to lie in the same horizontal plane as the robotic arm. A 43 cm (length) × 33 cm (width) × 0.30 cm (height) touchscreen panel (Keytec Inc., Garland, TX, USA), with a down sampled resolution of 1024 × 768 pixels was horizontally mounted 2.5 cm above the robotic arm, more specifically the screw head where participants placed their thumb. The touchscreen was used to record localization endpoints, made with the left hand, to proprioceptive hand-targets; the felt location of the right thumb resting on top of the robot handle, which was just under the touchscreen. The lights were dimmed, and the subject’s view of their training (right) arm was blocked by the reflective surface and a black cloth draped over their right shoulder. The view of the untrained left hand was not concealed, but lit by a small lamp, so that the left arm was visible during the proprioceptive localization task. This procedure ensured any errors in localizing the unseen right target hand could not be attributed to errors in localizing the left, reaching hand. The experimental paradigm used in-house tcl code running on a Linux PC, interfacing with the robot manufacturer’s API to control the robot and read-out it’s position at 50 Hz. The touch screen was connected to a separate Windows PC running a custom C-based server that provided position information over an HTTP socket to the tcl experiment.

Visual Targets: The targets were located radially, 12 cm from the home position at 60°, 90° and 120° in polar coordinates, each represented by a 1 cm diameter yellow circle (Fig 1B). Fig 1B–1D displays the different tasks and target locations used throughout the experiment. The cursor, used to represent the subject’s hand, was a green circle 1 cm in diameter. The home position was visible only briefly before the target onset and to guide participants back to the home position during two of the three tasks, it was located ~20 cm in front of the subject at their body midline. The home position and the target were never shown at the same time. The intertrial interval, where participants’ right adapted hand was locked at home, lasted 500 ms.

Proprioceptive Stimuli: For proprioceptive localizations, the right hand served as a target, and it was passively moved by the robot to one of the three target locations previously described. A beep then signaled for participants to use their left untrained hand to indicate on a touchscreen the felt location of their trained right hand underneath the touchscreen (Fig 1D). Once the touchscreen registered their touch, the right target hand was allowed to move freely back to the home position along a robot-constrained path [9,23], while only the home position was visible. The hand was then locked at the home position for 500 ms before it was passively moved to the next target site.

### General procedure

**Fig 2:**
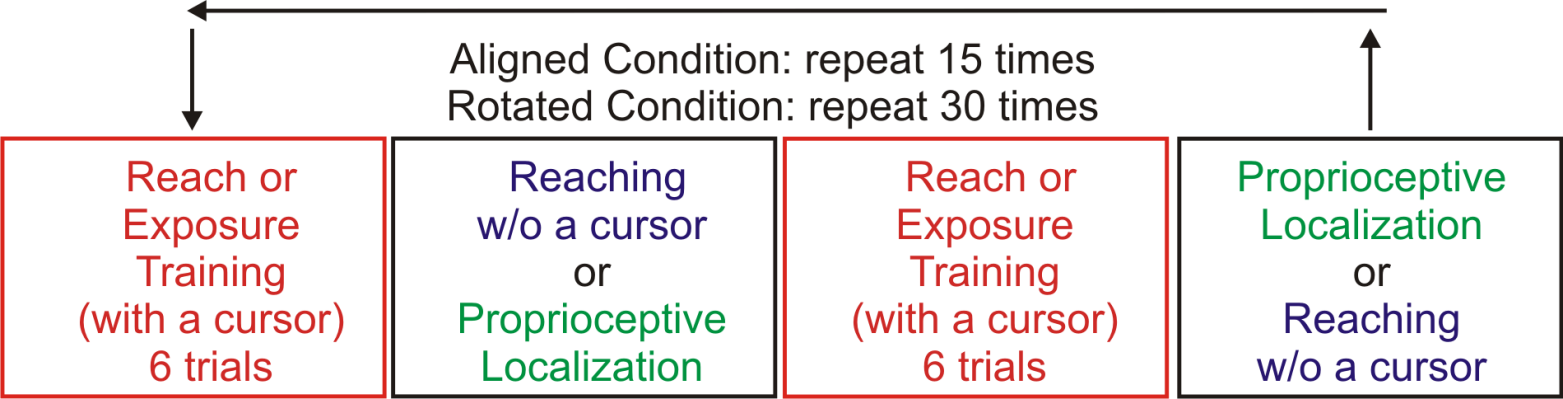
Testing session breakdown. Participants completed three tasks under two separate conditions that include exposure training with an aligned cursor and with a cursor rotated 30° CW. As shown by the four boxes, each block consisted of 18 trials including two sets of 6 exposure training trials, alternatingly followed by 3 no-cursor or 3 localization trials. This amounted to a total of 270 (18 trials X 15 iterations) trials during the **aligned condition** and 540 (18 trials X 30 iterations) trials during the **CW rotation condition**. All participants completed the same tasks throughout training, but the order in which the tasks were completed was counterbalanced across participants with two versions of task order **(version 1 and version 2)**.

To allow for direct comparison to a previous study using classic visuomotor rotation training, the procedures here match that of the first day of training [22]. The experiment was comprised of an Aligned and Rotation condition, each of which consisted of a repeated series of three tasks (Fig 2). The Aligned condition was used to collect baseline measures while training with a cursor whose motion was aligned with the constrained hand movement. The Rotation condition began abruptly and introduced a 30° CW discrepancy between the direction of cursor motion and robot-generated hand path during training.

#### Exposure training

Participants began each session with six exposure training trials (Fig 2 red text). Before each set of six trials the word “Cursor” appeared for 1000 ms to indicate the type of subsequent task. After this, a target appeared for only 250 ms then disappeared, and participants moved their hand along the robot-generated constrained path in order to move the cursor toward the remembered target site. Unlike during traditional visuomotor rotation training, the robot constrained the hand motion so participants could only move their hand forward and back along a straight path, much like a force channel. If they attempted to move outside of the pathway, a resistant force, proportional to the depth of penetration with a stiffness of 2 N/mm and a viscous damping of 5 N/(mm/s), was created perpendicular to the pathway [28]. The green cursor, representing their hand, moved radially out with the hand, but always went straight to the intended target. Once the participant moved at least 9 cm out and held their hand still for 250 ms, the green cursor turned red signaling the end of the trial. The hand returned to the home position along the same robot generated path at a speed of 4 cm/s. It was then held in place at home for 500 ms, when the next trial began.

We refer to this task as exposure training as participants did not freely generate their movement nor decide the direction of their movement. By using a constrained path, participants determined how far and fast they moved their hand along the path, while being *exposed* to a discrepancy between visual and proprioceptive feedback on their movement. Thus exposure training, by greatly restricting self-generated movements, eliminates visuomotor error signals to isolate the effects of the visual-proprioceptive discrepancy of the hand on motor and sensory changes associated with learning. In previous studies on exposure training conducted in our lab we used an entirely passive paradigm, the hand was dragged and participants made no movements. The results were consistent whether participants experienced entirely passive training or this “semi-active” exposure training used here [9]. We chose to use the semi-active exposure training paradigm here to reduce experimental time and increase the comfort of the participants. For the aligned cursor exposure training, sets of 12 trials were repeated 15 times for a total of 180 trials. In the rotated cursor exposure training, these sets were repeated 30 times for a total of 360 trials.

In order to ensure participants were looking at the cursor during exposure training, as in our previous studies [5,9] we had the cursor blink on 50% of all trials for 33.3 ms, along the middle half of the cursor motion. At the end of the trial, participants reported if the cursor blinked or not by pressing one of two keys with their left hand. A single beep was given when participants gave the incorrect answer, e.g. saying it did not blink when it did or vice versa. Participants were correct ~88% of the time, so all were included in the subsequent analyses.

#### Reaching without a cursor

Reaching without a cursor before and after training with a rotated cursor is a common method for measuring reach aftereffects (Fig 2 purple text). But for this study, we wanted to measure the time course of these deviated movements, so we sampled every six trials throughout both training sessions. More specifically, after completing at least six exposure training trials participants completed three no-cursor reaches, one to each of the same three targets as during exposure training. This task was very different from the exposure training trials as the subjects had full control over their movements and were not constrained. In addition, they received no visual feedback of their reach. The words “No-Cursor” were displayed for 1000 ms before each block of three trials. The participants were required to reach to the target, stop when they believed they had achieved the target, and hold their hand still for 250 ms to complete the trial; receiving no feedback on how close their reach was to the target. At that time, the target disappeared and the home position appeared and participants returned to the home position (along a robot-generated path). During aligned-cursor training, there were 15 iterations of 3 trials (45 no-cursor reach trials in total), while during the rotated-cursor training conditions, there were 30 iterations or 90 no-cursor trials in total.

#### Proprioceptive localization

The proprioceptive localization task differed from the previous two tasks in that there was no visual stimulus (no visible target; Figs 1D and 2, green text). Participants were instructed with the words “Reach to Hand” for 1000 ms before each block of three trials. During proprioceptive localizations the trained right hand was used as the target, which was moved by the robot to one of the three target locations. This passive movement of the hand took 650 ms to cover the 12 cm distance. A beep signaled participants to use their left index finger, on a horizontal touchscreen (Fig 1A and 1D), to indicate the felt location of the unseen right target-hand, more specifically the thumb, resting on top of the handle. Once the touchscreen response was registered, the robot released the right target hand and the home position reappeared. During aligned cursor training, there were 15 iterations of 3 trials or 45 proprioceptive localization trials in total. During the rotated cursor training condition there were 30 iterations or 90 trials in total.

We interleaved these three tasks to get a clear picture of the rate of sensory and motor changes that arise during repeated exposure to a visual-proprioceptive discrepancy. Exposure training was always the first task, but to counterbalance task order, half our participants completed three no-cursor trials immediately after the first set of training (version one), whereas the other half immediately completed three proprioceptive localizations (version two). All participants then completed six more training trials and then the one remaining task (Fig 2). The entire experiment took approximately 75 minutes to complete.

### Data analysis

The first goals of this study were to characterize the rate or time course of changes in reach aftereffects and proprioceptive recalibration when participants were exposed to a visual-proprioceptive discrepancy, i.e. without making voluntary reaching movements. Participants did not make volitional reaching movements, since their hand motion was directionally constrained, so we used reach errors during no-cursor reaches, or “reach aftereffects” to quantify motor learning. Changes in hand localization were used to measure proprioceptive recalibration. To better estimate the overall motor and sensory changes, we analyzed results across three sets of “Blocks”: each block or time point analyzed was an average of three trials. These averages came from the final three aligned trials, the initial and the final three rotated trials.

#### Proprioceptive recalibration

The analysis of proprioceptive changes used the angular endpoint error as provided by the difference between robot-guided endpoint of the unseen, trained hand and the responses on the touchscreen. We use these localization endpoints to make the measure comparable to the no-cursor reach trials.

#### Reach aftereffects

To determine if participants altered their reaches as a result of exposure training with the 30° CW rotation, we measured reach endpoint errors when receiving no visual feedback of hand location (no-cursor trials). The reaching error is calculated based on the angular deviation between the reach endpoint and the target location, relative to the home position. Reach aftereffects refer to the changes in these no-cursor reach endpoints during rotated-cursor exposure training relative to baseline no-cursor reaches.

#### Comparison to classic training

Another goal was to compare the results collected in this study (n = 19) to those collected from a previous study with a very similar procedure, with the exception of the training task (n = 20) [22]. The training paradigm used in [22] was the traditional or classic visuomotor adaptation training which allowed participants full control over hand movements. This classic visuomotor training involves both sensorimotor error-signals and visual-proprioceptive discrepancy signals to drive sensory and motor changes during adaptation. In the current study, we used exposure training where the participants were not able to choose the direction of movement and thus received no error signal from movement discrepancy. Apart from this the trial schedule was exactly the same, allowing comparison of the effect of the training types on no-cursor reach errors and proprioceptive estimates of hand location.

#### Statistical analysis

Reach aftereffects, proprioceptive recalibration and the similarities across training types were analyzed using identical procedures. In order to assess the progression of reach aftereffects (change in no-cursor reaches) and change in hand proprioception during exposure training we conducted a one way ANOVA to examine the effect of Block (three levels, aligned final, rotated initial and final) on these changes. We then tested how they differ across training with a Mixed-ANOVA that included the training type (classic vs. exposure) in addition to Block for both proprioceptive localizations and no-cursor reaches (separately). With the exception of the comparison of baseline results between the two training groups and between the baseline and the block after the initial 6–12 reach-training trials (initial block), all other planned follow-up comparisons were baseline-corrected, in that the results from the final aligned block were subtracted out. To correct any family-wise error for these follow-up comparisons (t-tests) we report Bonferroni corrected p-values, so as to still use a p-criterion of .05. This correction was completed using the p.adjust function and reported effect sizes (Cohen’s d) were calculated in R. Data preprocessing and statistical analysis was completed implemented R version 3.4.4 [29], using the package ‘ez’ for ANOVAs (Lawrence, 2016).

## Results

### Reach aftereffects

**Fig 3:**
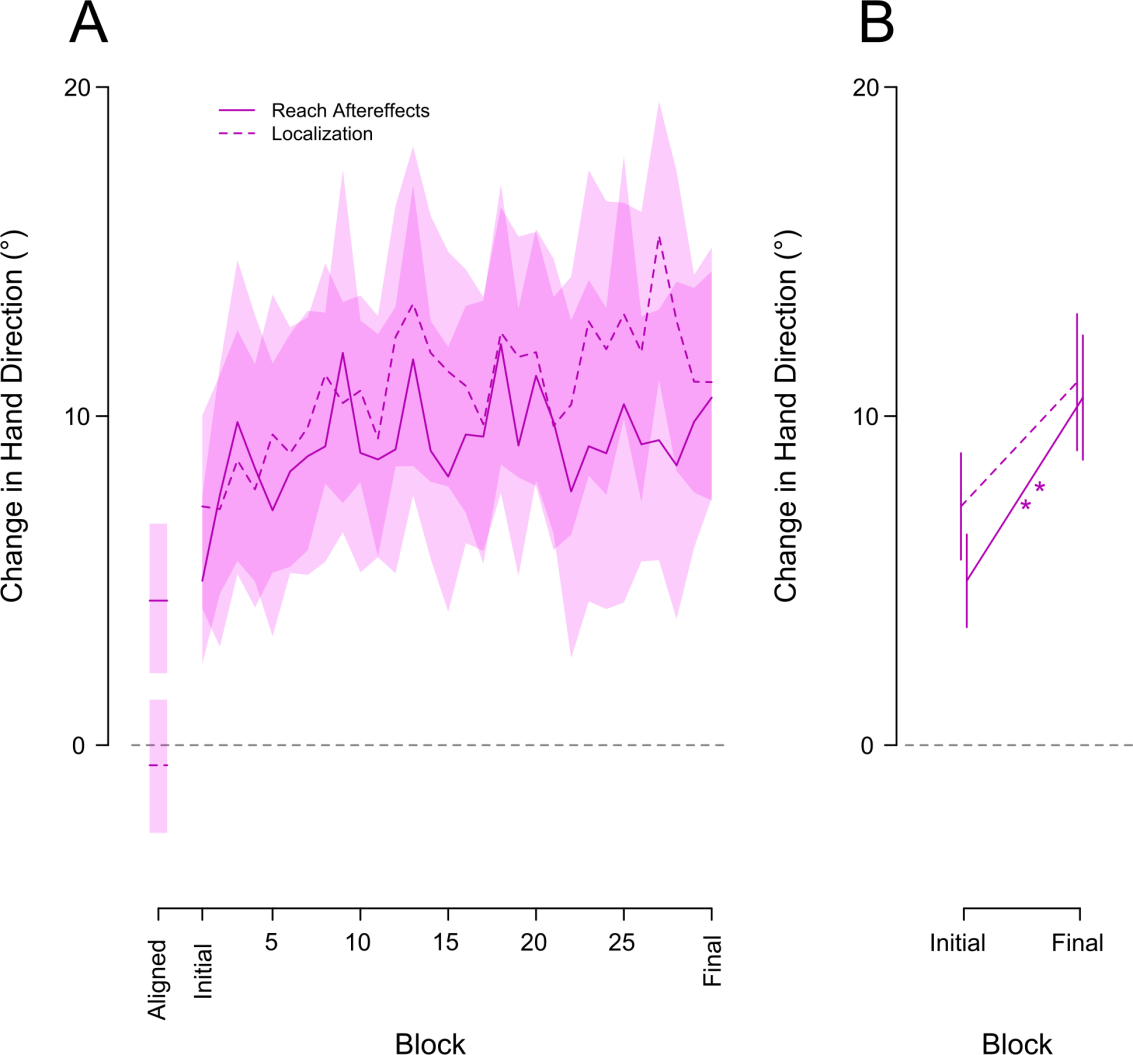
Reach aftereffects (solid lines) and proprioceptive localizations (dashed lines) plotted as a function of all blocks (A) and for the first and last block (B) during the rotated condition. No-cursor reaches and proprioceptive localization for the final block of the aligned condition are also shown in at the far left of panel **A**, the rotated condition results have these baseline data subtracted out to normalize the results. The shaded areas for the curves in **A** represent a 95% confidence interval. The asterisk in **B** indicates significant differences between blocks; one star is significant to .01, two stars are significant to .001. Error bars are +/- 1 standard error.

To gauge the effect of the visual-proprioceptive error signal on the time course of motor changes, we frequently measured reach aftereffects, from no-cursor reaches, throughout exposure training (see Fig 3). Participants completed three no-cursor reaches every 12 training trials. They were fairly accurate when completing this task in the aligned condition, with a bias of 4.39° averaged across all 19 subjects. After exposure to the misaligned cursor subjects showed significant reach aftereffects [Aligned vs. initial vs. final: F_(2,36)_ = 5.48, p = .008]. The solid purple curve in Fig 3A displays the time course of reach aftereffects across all blocks of misaligned exposure, while the solid line in Fig 3B shows the same for only the first and final of these blocks. Only 6 or 12 visual-discrepancy trials were necessary to create significant changes in motor output with reach aftereffects of 5° [aligned vs. initial block: t_(18)_ = -3.544, p = .004, d = -1.14]. These reach deviations continued to significantly increase following another 168–174 training trials by 5.57° [initial vs. final: t_(18)_ = -3.806, p = .002, d = -1.22]. Relatively quickly participants showed significantly deviated no-cursor reaches following exposure training.

### Proprioceptive recalibration

Subjects were quite good at locating their unseen hand during veridical training, falling on average within a degree of the target site (.9°). As expected their felt estimate of the unseen hand position shifted significantly during passive exposure to the cursor-hand discrepancy [Aligned vs. initial vs. final: F_(2,36)_ = 16.43, p < .001]. The dotted purple curves in Fig 3A illustrate this change in proprioceptive localization across all of the blocks of misaligned visual-proprioceptive exposure, while Fig 3B shows the same changes for only the initial and final block of exposure. When comparing the final aligned trials to the initial block of trials completed during this misaligned training, subjects’ felt hand position was already shifted by 7.5° [aligned vs. initial: t_(18)_ = 4.4807, p < .002, d = 1.44]. This shift continued to increase across subsequent training trials to 11.32° but this did not quite meet significance-threshold [initial vs. final: t_(18)_ = 2.421, p = .052, d = .78]. In summary, experiencing a visual-proprioceptive mismatch of the hand is enough to produce significant shifts in felt hand position after only six such exposure trials.

### Classic training vs. exposure training

**Fig 4:**
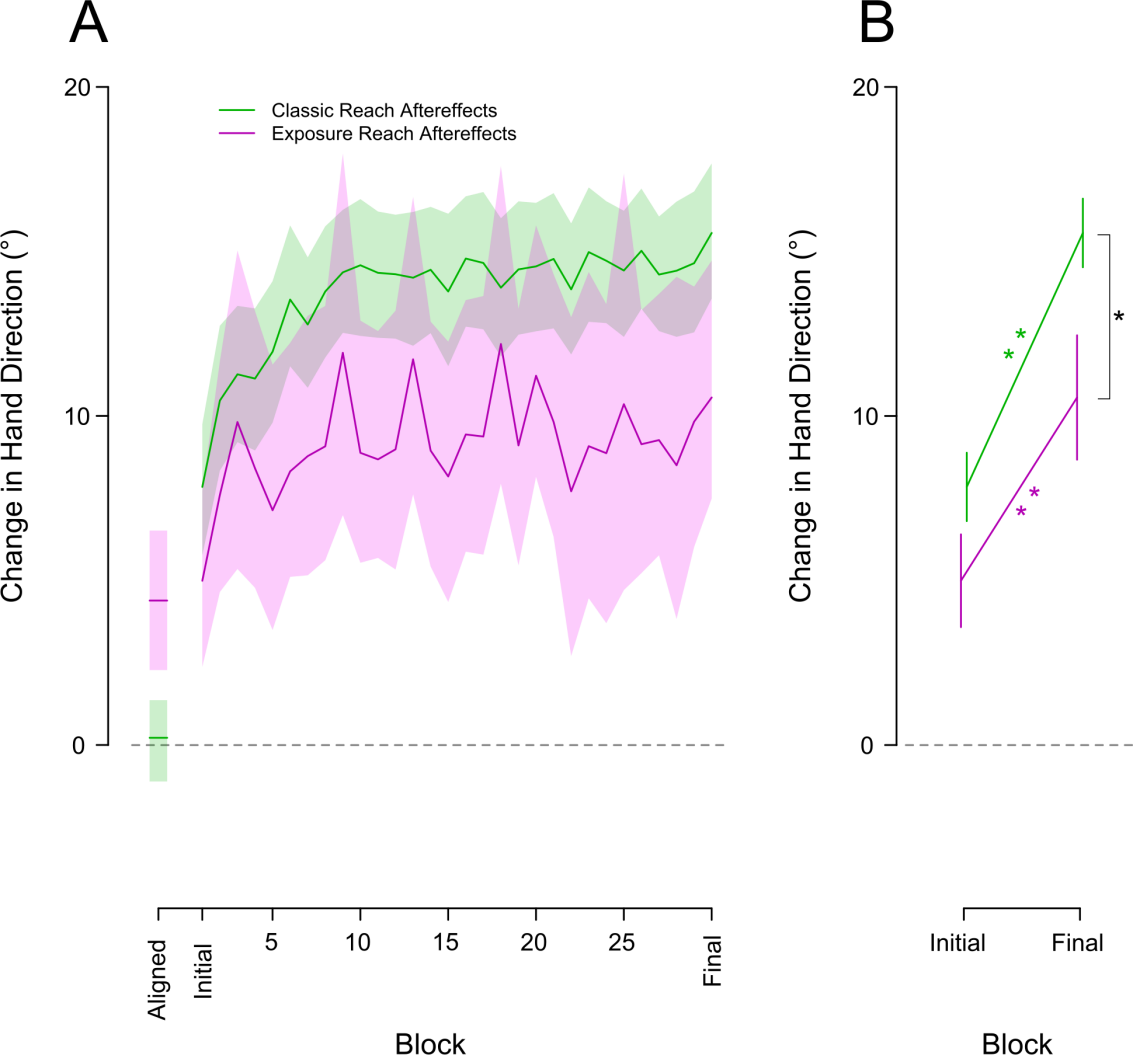
Reach aftereffects across training types. Mean change in no-cursor reaches relative to baseline for all blocks (A) and just the first and final block (B) of the classic (green) and exposure (purple) rotated conditions. As in Fig 3, the final block of the aligned condition is also shown in the far left on panel A and the rotated conditions have the aligned baseline subtracted. **A:** The solid lines within the coloured curves represent the block means while the coloured areas represent a 95% confidence interval. In **B,** the asterisk indicates significant differences between blocks; one star is significant to .01, two stars to .001. The error bars are +/- 1 standard error.

**Fig 5:**
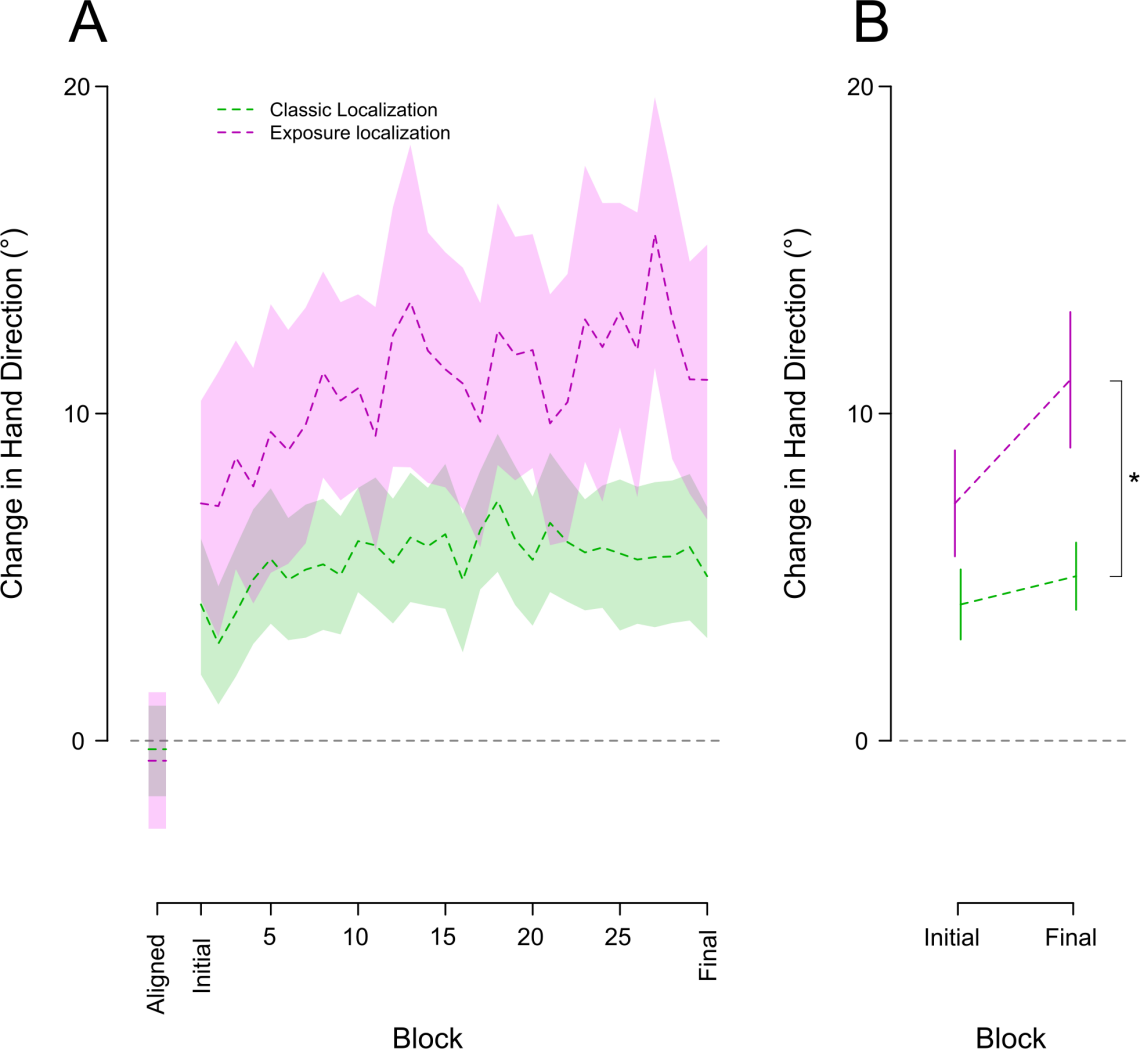
Change in proprioceptive localizations across training types. Mean change in hand proprioception relative to baseline for all blocks (A) and just the first and final block (B) of the classic (green) and exposure (purple) rotated conditions. As in Fig 3, the final block of the aligned condition is also shown in the far left on panel A and the rotated conditions have the aligned baseline subtracted. **A:** The dashed lines within the coloured curves represent the block means while the coloured areas represent a 95% confidence interval. In **B,** the asterisk indicates significant differences between blocks; one star is significant to .01, two stars to .001. The error bars are +/- 1 standard error.

Our final goal was to compare the rate of proprioceptive and motor changes following exposure training to the rate of the same changes produced during classic visuomotor adaptation to a 30° clockwise cursor rotation [22]. Again, the trial schedule for the two experiments was identical. A two way Mixed ANOVA with a 3 level within factor of block (final block of aligned, initial and final block of rotated) and a 2 level between subjects factor of training type (classic versus exposure training) was conducted on both measures of interest, reach aftereffects (Fig 4) and proprioceptive localizations (Fig 5). The results of the main effect of block for exposure training are stated above and were both significant. There was also a significant interaction between the factor of block and training type for both measures [reach aftereffects Block X Training type: F_(1,74)_ = 7.73, p < .001]. Fig 4A shows reach aftereffects during classic visuomotor training in green and for exposure training in purple. The interaction described above may be partially driven by a significant difference in the direction of no-cursor reaches produced during aligned-cursor training in both experiments [Aligned: t_(37)_ = -3.215, p = .006, d = -1.03]. Despite this difference in baseline no-cursor reaches, the significant change in these no-cursor reaches following a single first block (6–12 trials) of the rotated training did not differ between the two training types [Initial: t_(37)_ = -1.581, p = .357, d = -.51]. These initial reach aftereffects were 5° for exposure training and 8° for classic visuomotor training. Following an additional 324–330 training trials those who experienced exposure training exhibited reach aftereffects of 10.5°. As expected, those who experienced classic training had reach aftereffects much larger than the exposure training group 15.5° [Final: t_(37)_ = 2.503, p = .045, d = .8]. These results suggest that training that engages the motor system leads to larger aftereffects, although exposure training still leads to considerable motor changes.

Proprioceptive localizations showed a fairly similar pattern of change across training types (green curves in Fig 5) with a significant interaction between the factors block and training type in the above described 2 X 3 Mixed ANOVA [F_(1,74)_ = 3.08,p = .05]. This interaction was not driven by any difference in the significant changes between the aligned block and initial block for the two training groups (p>0.05). For both training types, only 6–12 rotated training trials lead to a clear and significant shift in proprioceptive estimates of hand position of 7.6° for exposure training and 4.2° for classic visuomotor training. The interaction instead was likely driven by the differences in proprioceptive recalibration in the final block of training for the two groups [Final: t_(37)_ = 2.910, p = .005, d = .93]. Those in the exposure training group had shifted their felt hand position by 11.3° whereas those in the classic training group had only shifted by 5.09°. Thus, while the changes are unexpectedly larger when training with only a visual-proprioceptive discrepancy than when training with both the sensory discrepancy and visuomotor error signals, the main point here is that the proprioceptive recalibration in both cases emerges quickly and follows a similar time course.

## Discussion

Here we show that without any volitional movements and only exposure to a discrepancy between seen and felt hand location, motor adaptation and proprioceptive recalibration occur rapidly. This indicates that visual-proprioceptive discrepancies of hand motion are sufficient training signals to produce the sensory and motor changes associated with model-based motor learning. Moreover, the time course of the changes were similar to that during classic visuomotor adaptation training, where the changes can also be driven by traditional error-based signals [22]. The similar rate of change produced during both exposure training and classic training suggest that a substantial part of the motor and nearly all of proprioceptive changes that are produced with classic training may be driven by visual-proprioceptive discrepancies. This in turn suggests that visual-proprioceptive discrepancy is a critical training signal which can account for much of the changes associated with visuomotor learning.

### The effect of being exposed to visual-proprioceptive discrepancies

Despite the absence of volitional movements and hence their efference copies and ensuing predicted sensory consequences, exposure to a visual-proprioceptive discrepancy of the hand leads to substantial initial reach aftereffects that grow larger with continued training. While a few studies have measured reach aftereffects following this type of passive training, this study is the first to reveal how quickly these reach aftereffects can emerge, i.e., after merely 6 or 12 trials of exposure training. By the end of exposure training, reach aftereffects double in magnitude, to ~10°, or 33% of the sensory discrepancy. This is comparable to other studies [5,9,10,25], that typically find reach aftereffects after exposure training to be 30–60% of the size of those following classic visuomotor rotation training. Reach aftereffects have also been shown to scale with the size of the distortion for classic visuomotor rotation training [14], but not for exposure training [5]. Thus, while reach aftereffects emerge for both types of training, the distortion-dependent increase in extent is not consistently present. Here we show how quickly reach aftereffects emerge during exposure training, and that the extent of these motor changes differ as a function of the two types of training, which likely reflects the different contributions of visuomotor errors and visual-proprioceptive discrepancies.

To an even greater extent, this exposure training based on a visual-proprioceptive discrepancy only, leads to a quick and substantial change in proprioceptive estimates. Proprioceptive estimates of hand position were recalibrated by 7.5° after 6–12 rotated training trials. Moreover, further exposure training continued to shift these estimates to 11.3° or almost 40% of the visual-proprioceptive discrepancy. This final proprioceptive recalibration is similar to that found in other exposure-training studies [5,9,10,25], but it is larger than in the classic training version of the same paradigm where it was 5.09° or 17% of the rotation [22]. Usually, the size of change in felt hand position is similar to that produced following classic training, ~15–40% of the distortion [4,9,10,14,25]. We are not sure why we find a larger magnitude of recalibration in this case, but since both are within the expected range the differences might be attributed to idiosyncratic group differences. While the different asymptotic levels of change may be due to irrelevant differences between the participants, the time course of the change is not affected by this. This suggests that we can be confident that these proprioceptive changes are caused by the visual-proprioceptive discrepancy training signals in both groups.

### Time course of changes during exposure and classic training

The few studies investigating the time course of sensory and motor changes during adaptation to a visual or dynamic perturbation [16,21,22] all use the classic training paradigm where participants generate their own reaching movements to the target. The first two such studies showed significant sensory changes in felt hand position or motion only after ~70 training trials when using a 2-AFC method [16,21]. This 2-AFC procedure takes 40+ trials to measure a single point and could potentially suffer from decay so that the first few measurements failed to find any change in proprioceptive estimates. Using our quicker method of measuring hand proprioception, we find that proprioceptive changes emerge after just 6 or 12 trials for both classic visuomotor training [22] and exposure training with the rotated cursor. The extremely rapid change in proprioceptive recalibration we observe in both of our studies suggests that these somatosensory changes are not only robust but are quite sensitive to visual-proprioceptive discrepancies.

The speed with which reach aftereffects arise during classic visuomotor adaptation has also only recently been investigated [21,22]. In both of these studies, significant reach aftereffects emerged within 5–6 reach-training trials with a 30° cursor rotation. Again, in the current study, we find the same time course in motor changes during exposure training even though the motor system is not engaged in this type of training. Interestingly, both previous studies found reach aftereffects do not continue to increase after ~40 reach training trials. Here, we also find this same plateau in reach aftereffects during exposure training. This same saturation has been seen in many studies that include prolonged training [14]. Here, with exposure training, reach aftereffects develop and plateau in a similar pattern to that produced when participants make volitional reaches with a visible cursor that is rotated (i.e. “classic” reach training), albeit with a smaller asymptote. This indicates that visual-proprioceptive discrepancies directly inform motor systems.

As seen above, the time course of change in proprioceptive estimates of hand location differs from that of reach aftereffects in both types of training. There are substantial initial changes in both measures with only reach aftereffects significantly increasing with further training, while the change in hand localization is immediately close to asymptote. This difference in the time courses suggests that the neural processes underlying proprioceptive recalibration may be different from those producing changes in reach aftereffects and visually guided reaches. Furthermore, seeing as the rate of changes produced during exposure training are similar to those during classic training, we can conclude that a substantial part of the changes seen with classic training are driven by visual-proprioceptive discrepancies. As alluded to above, 30–60% of reach aftereffects are likely driven by the visual-proprioceptive discrepancy, which appears to also partly drive motor learning as a whole.

### Potential neural basis

Both classic and exposure training have been used with cerebellar patients to help identify possible brain areas associated with movement and sensory error signals. When healthy older adults and mild cerebellar ataxic patients were tested on both classic and exposure training with a gradually rotated cursor [10], the two groups produced similar shifts in proprioception with reach aftereffects being smaller, mimicking previous studies. These results suggest that the cerebellum may not be required for proprioceptive recalibration and that this process may be occurring in another brain area, such as the posterior parietal cortex (PPC) [7,10,30–32]. The PPC has been shown to be involved in state estimation [33,34], as it receives multisensory information, making it a likely candidate for involvement in proprioceptive recalibration. This is consistent with our working hypothesis that proprioceptive recalibration is likely due to mechanisms, and brain areas, that differ from those involved in motor changes such as reach aftereffects which tend to depend more on the cerebellum.

### Conclusion

Using exposure training, we found that changes in reach aftereffect and proprioceptive hand localization emerge after only 6 exposure-trials and follow a time course similar to that found during classic visuomotor adaptation. This suggests the visual-proprioceptive mismatch plays a large and unique role in both the early rate and the extent of adaptation. These different time courses also suggest that reach aftereffects and proprioceptive recalibration are driven by different mechanisms and most likely different brain areas. In sum, we show here that visual-proprioceptive discrepancies lead to very rapid proprioceptive recalibration and make a substantial and very fast contribution to motor learning and reach aftereffects.
